# Development and validation of a brief three-item form of the perceived social support questionnaire (F-SozU K-3)

**DOI:** 10.1016/j.ijchp.2024.100496

**Published:** 2024-08-30

**Authors:** Julia Petersen, Anna C. Reinwarth, Manfred E. Beutel, Elmar Brähler, Oliver Decker

**Affiliations:** a Department of Psychosomatic Medicine and Psychotherapy, University Medical Center of the Johannes Gutenberg-University Mainz, Untere Zahlbacher Straße 8, Mainz 55131, Germany; b Department of Medical Psychology and Medical Sociology, Medical Faculty, University of Leipzig, Leipzig, Germany; c Else-Frenkel-Brunswik Insitute for Democracy Research in Saxony, University of Leipzig, Leipzig, Germany; d Sigmund-Freud University Berlin, Berlin, Germany

**Keywords:** Social support, Brief measure, Large scale surveys, Psychometric properties, Norm values

## Abstract

**Objective:**

The purpose of the present study was to develop and validate a brief screening instrument (F-SozU K-3) for the measurement of perceived social support in large scale surveys by shortening a well-established German questionnaire (F-SozU K-6).

**Method:**

First, a brief three-item version of the F-SozU was developed based on a representative sample of *N* = 2482 respondents using exploratory and confirmatory factor analysis. Second, the newly developed brief three-item questionnaire was evaluated and standardized in an independent second representative population sample (*N* = 2501).

**Results:**

A suitable three-item solution with a good internal consistency (α = 0.89, ω = 0.89) was identified. Full invariance across sex and partnership was established. Construct validity of the brief three-item form was established. Younger age, female sex, partnership status, and current employment were positively associated with higher social support scores. Norm values for the general sample and separately for sex and partnership status were reported.

**Conclusions:**

The newly developed F-SozU K-3 is a reliable and valid screening instrument. It can be used as an economical alternative to previous longer instruments, especially in large scale surveys.

## Introduction

Decades of research have highlighted the important role of social relations in preserving physical and mental health (Holt-Lunstad et al., 2010; Kawachi, 2001; Kuiper et al., 2015; Lakey & Orehek, 2011). Most recently, the role of social relations has increasingly come to the forefront of research and public health following discussion about psychosocial consequences of the public health measures during the COVID-19 pandemic on mental health (e.g., social isolation and loneliness) (Ernst et al., 2022; Holt‐Lunstad, 2021).

Despite the numerous definitions and methods for measuring social relations, research consistently identifies three main dimensions: the extent of social integration within one's social networks, the functional social support received through supportive interactions, and the perceived social support, which refers to an individual's subjective beliefs and perceptions regarding the availability of support (Cohen et al., 2000). A meta-analytic review analysing the effect of the three conceptualizations on mortality, highlighted the importance of the functional aspects of social relations. The authors concluded that the magnitude of influence of received and perceived social support on risk for mortality is comparable with well-known risk factors (e.g., obesity, smoking, alcohol consumption) (Holt-Lunstad et al., 2010). Moreover, the essential role of perceived social support in preventing mental health disorders (e.g., suicidal ideation (Otten et al., 2022)) has been documented. Thus, developing and maintaining meaningful social relations can be significant focal points in the development and promotion of mental health.

For the use in epidemiological studies where time, costs and effort are crucial, valid brief screening tools for social support are needed as alternatives to many scales with a large number of items developed in the last decades (e.g., the MSPSS (Multidimensional Scale of Perceived Social Support (Kazarian & McCabe, 1991)) or the PSSS (Perceived Social Support Scale (Procidano & Heller, 1983))). Moreover, recently used short measures have not previously been tested in the general population or were characterized by low reliability and validity (e.g., short forms of the OSSS (Oslo Social Support Scale (Naeem et al., 2008; Van Lente et al., 2012))). For assessment of social support in German-speaking populations, the Social Support Questionnaire (Fragebogen zur sozialen Unterstützung; F-SozU) developed by Fydrich et al. (1999) is an accepted and valid measure which conceptualized social support in a functional way as perceived social support from one's social network (Barrera, 1986; Heller & Swindle, 1983; House, 1987). Fifty-four statements regarding generalized experiences of perceived or anticipated social support are rated on a five-point Likert scale, ranging from 1 (does not apply) to 5 (exactly applicable). The original 54-item version of the F-SozU and its 22-item version (F-SozU K-22 (Fydrich et al., 2007)) assesses the three central dimensions of perceived or anticipated social support which can be summarized to a total score of the extent of one's general perceived or anticipated social support: instrumental support (e.g., practical help with daily problems), emotional support (e.g., experiencing consolation), and social integration (e.g., belonging to a social network). In addition to the 54- and 22-item version, Fydrich et al. (2009) developed a 14-item version of the F-SozU (F-SozU K-14) assessing general perceived social support. Kliem et al. (2015) developed and standardized the most recent six-item version (F-SozU K-6) using representative German population samples. In line with the recommendation for the interpretation of the F-SozU K-14, a unidimensional solution is suggested. The F-SozU K-22, F-SozU K-14 and F-SozU K-6 showed good reliability. Perceived social support assessed by the original 54-item version of the F-SozU and its shorter versions has been found to be associated with better social competence, and less social insecurity, depression, anxiety, as well as somatic symptom strain, and several sociodemographic factors (e.g., sex, education, marital status) (Fydrich et al., 2007; Kliem et al., 2015).

Currently, the F-SozU K-6 is widely utilized in both national and international research for assessing perceived social support. Notably, within a diverse array of studies, including one conducted in a representative German population, findings underscored its significance in understanding resilience. In particular, lower levels of perceived social support were predictive of somatic symptoms, and correlated with higher levels of distress and instances of adverse childhood experiences (Beutel et al., 2017b). In the context of exploring the mental health ramifications of the pandemic, researchers, such as Sommerlad et al. (2022), adapted the F-SozU K-6 for their investigations. Amidst the COVID-19 lockdown, their findings indicated a clear association between heightened perceived social support and reduced symptoms of depression among residents of the UK. Moreover, its reliability across different cultures and its consistent measurement invariance were confirmed through psychometric evaluations across various populations, including Chinese, German, Russian, and U.S. samples, affirming its utility for cross-cultural epidemiological studies (Lin et al., 2019). Nevertheless, for large scale surveys with constraints of cost, time and space a short screening instrument measuring perceived social support with high reliability and validity is desired. To the best of our knowledge, to date, no shorter versions than the F-SozU K-6 have been developed and validated.

The present work aimed to develop and validate a brief screening instrument (F-SozU K-3) for the measurement of perceived social support in large scale surveys by shortening the well-established German questionnaire (F-SozU K-6) in two phases. Our aims were to:

(1) evaluate the six items of the F-SozU K-6 and develop a three-item version, the F-SozU K-3
(2) assess the psychometric properties of the F-SozU K-3, including its reliability, factorial structure invariance across sex and partnership status, as well as construct validity and item characteristics
(3) report norm values regarding partnership status and sex of a representative sample of the German population.

## Method

### Participants

For the present study, we used data from two nationwide, representative (in terms of age, sex, and educational level) German population surveys using the same methodology. The survey of phase 1 (Sample 1) was conducted in 2013. The second survey (Sample 2) was conducted in 2018. Both surveys were conducted as face-to-face household interviews conducted by trained interviewers, who were employed by the independent market research institute USUMA. Participating households were selected following a random route procedure combined with Kish selection (Kish, 1949). The inclusion criteria for participation were an age of ≥ 14 years and an adequate understanding of the German language.

Participants were provided with detailed information about the study's procedures, data collection, and anonymization of personal data prior to data analysis. The participants’ verbal informed consent to participate in the study was then obtained and acknowledged by the interviewers. The Ethics Commission of the Medical Faculty of the University Leipzig approved the studies contents and procedures, including the consent procedure (Sample 1: 050/13‐11,032,013, Sample 2: 132/18‐ek). In addition to ICH-GCP-guidelines, both surveys adhered to the guidelines outlines in the ICC/ESOMAR International Code of Marketing and Social Research Practice as well as the Declaration of Helsinki.

We excluded participants with missing data for the F-SozU K-6 in both surveys (*N* = 26 in Sample 1 and *N* = 15 in Sample 2) from our analysis, resulting in an analysis sample of *N* = 2482 participants with 46.9 % men and 53.1 % women with a mean age of 49.62 years (*SD* = 18.31) for the development of the F-SozU K-3 (Sample 1). Sample 2 comprised data of *N* = 2501 participants with 45.5 % men and 54.5 % women with a mean age of 46.01 years (*SD* = 17.58).

### Instruments

*Social support:* Social support was initially assessed using the brief form of the Perceived Social Support Questionnaire (F-SozU K-6) (Kliem et al., 2015) consisting of six items in Sample 1. This questionnaire covers general aspects of perceived social support, e.g., “When I am sick, I can ask friends/relatives to handle important things for me without hesitation.” Participants were asked to evaluate each item on a five-point Likert scale, ranging from 1 (does not apply) to 5 (exactly applicable). Answers are summarized to a sum score (6–30), with higher scores indicating higher levels of perceived social support. The F-SozU K-6 of this sample showed a very good internal consistency (*α* = 0.89, *ω* = 0.91). In the second sample, only the three-item version of the F-SozU was used.

*Sociodemographic characteristics:* Assessed were demographic factors such as age, sex (male/female as identified by the interviewers) partnership and employment status, as well as household size. Additionally, the highest achieved school degree was surveyed and subsequently recoded in 0 (no high school degree) and 1 (having a high school degree). Further, we inquired about household income using 13 income categories: 1 = under 500€, 2 = 500–650€, 3 = 650–750€, 4 = 750–900€, 5 = 900–1000€, 6 = 1000–1150€, 7 = 1150–1250€, 8 = 1250–1500€, 9 = 1500–2000€, 10 = 2000–2500€, 11 = 2500–3500€, 12 = 3500–5000€, 13 = over 5000€. The equivalized income was calculated by first assigning the mean value of an income group to each respondent. Following this step, we divided this mean by the square root of the number of people living in the respondents’ household.

*Symptoms of depression:* The two-item Patient Health Questionnaire (PHQ-2) was used to assess depression symptoms. The PHQ-2 measures core depressive symptoms of depressed mood and anhedonia (Löwe et al., 2010; Wicke et al., 2022). Each item is scored on a scale from 0 (“not at all”) to 3 (“nearly every day”). Answers are summarized to a sum score (0–6), with higher score indicating higher levels of depressive symptoms. In the present sample, the PHQ-2 showed good internal consistency (ω= 0.82).

*Symptoms of generalized anxiety:* Symptoms of generalized anxiety were captured by the two-item Generalized Anxiety Disorder Screener (GAD-2) (Löwe et al., 2010; Wicke et al., 2022). Participants rated to what extent they were affected by the feeling of nervousness, anxiety, and the inability to stop or control their worrying over the last two weeks on a four-point scale from 0 (“not at all”) to 3 (“nearly every day”). Answers were added to a sum score (0–6), with higher scores indicating more symptoms of generalized anxiety. The GAD-2 has shown good internal consistency (ω= 0.81) in the present sample.

*Loneliness:* Loneliness was measured by a validated single item: “I am frequently alone/have few contacts”. Response options ranged from 0 (“no, does not apply”), 1 (“yes, it applies, but I do not suffer from it”), 2 (“yes, it applies, and I suffer slightly”), 3 (“yes, it applies, and I suffer moderately”), or 4 (“yes, it applies, and I suffer strongly”). In line with previous research, we recoded loneliness combining 0 and 1 to indicate "no loneliness or distress", 2 = "slight", 3 = "moderate", and 4 = "severe loneliness" in line with previous research (Beutel et al., 2017a; Reinwarth et al., 2023). Values ≥ 2 indicated “loneliness”.

### Procedure

Sample characteristics were reported as absolute numbers and percentages for categorical variables and as means with standard deviations for continuous variables. Internal consistency of the scores were estimated using Cronbach's Alpha (α) and McDonald's Omega (ω).

In order to determine a suitable, economic measure of perceived social support, we searched for a solution with high internal consistency, unidimensionality, and a small number of items. Preferably, the scale should contain one item for each of the three dimensions of the original scale. First, we tested which items demonstrated the highest correlations with each other. Next, we used exploratory factor analysis (EFA) and applied principal axis factoring to determine the optimal factorial solution. Three criteria were used to determine the optimal factor structure of the F-SozU K-3: Kaiser Guttman criterion, screeplot, parallel analysis. After we identified the suitable unidimensional three-item solution, we tested the internal consistency of the construct.

Next, we investigated the psychometric properties of the newly developed F-SozU K-3 using Sample 2. Selectivity was determined via item-total correlations. Item difficulty was calculated by dividing the sum of the values obtained by the maximum attainable item values. Factor validity was tested using confirmatory factor analysis. Maximum likelihood estimation was used since significant deviations from normal distribution were found. Chou et al. (1991) found this approach to be a valid and robust method in dealing with these types of deviations from normality. The goodness-of-fit was assessed via the Standardized Root Mean Squared Residual (SRMR), the Root Mean Square Error of Approximation (RMSEA), the Comparative Fit Index (CFI), and the Tucker Lewis Index (TLI). A model with a RMSEA and SRMR of < 0.05 indicates a good model, while values between 0.05 and 0.08 indicate reasonable fit. A CFI and TLI of >0.95 are indicators of good fit (Hu & Bentler, 1999; Van De Schoot et al., 2012).

Subsequently, factorial invariance was tested across sex and partnership status. We used Meredith and Teresi's (2006) sequential strategy as a guidance. In this strategy, configural, weak, strong, and strict measurement invariance were estimated individually and then subsequently tested against the stricter model. Configural invariance assumes, that there might be variations in the loadings, intercepts, and variances of the latent constructs among the different groups and therefore does not presume any restrictions. The weak invariance model constrains factor loadings to be equal across all groups. The strong measurement model additionally constrains item loadings, and, finally, the strict measurement invariance model constrains factor loadings, intercepts, and residual variances across groups. To assess the model fit, the most commonly used method is the chi-square test, though, it has been established that the accuracy of this test is largely influenced by sample size, potentially leading to a rejection of a reasonable model due to large sample sizes. Therefore, we relied on the abovementioned four indices goodness-of-fit assessment CFI, TLI, RMSEA, and SRMR. If the difference in CFI between the tested measurement invariance models is < 0.01, as proposed by Chen (2007), we can assume that the stricter tested measurement invariance is given.

Construct validity was estimated by using Spearman correlations. We tested the intercorrelations between the brief form of the F-SozU K-3 and depressiveness (PHQ-2), anxiety (GAD-2), and loneliness (GHS loneliness item). Further, group differences of the F-SozU-3 score between the age groups (14–49 vs. 50–91; groups were determined according to the median of 49), sexes (male vs. female), educational degrees (high school degree vs. no high school degree), having a partner (yes vs. no), and being currently unemployed (yes vs. no) were tested using *t*-tests. Effect sizes were reported using Cohen's d. Additionally, we calculated two-way ANOVAS with the factors sex and age, as well as the factors sex and partnership status.

Finally, we calculated norm values for the F-SozU K-3 in the German general population using percentiles. Those percentiles were calculated for the total sample and for subsamples based on sex and partnership status. For all statistical analyses, we used the software R version 4.1.2 and RStudio version 2021.09.2 (packages: dplyr (Wickham et al., 2023), psych (Revelle, 2023), lavaan (Rosseel, 2012)).

## Results

### Development of the F-SozU K-3

Information on the items of the F-SozU K-6 can be found in Supplementary Table 1. Correlations tests showed that the largest correlations could be found between items 2, 5, and 6, ranging from 0.65 to 0.71. Results from the EFA with these items indicated a unidimensional one-factor model; the Kaiser Guttman criterion had an Eigenvalue of 1.71 and explained 56.98 % of the variance. The scree-plot further confirmed the one-factor solution. Additionally, the parallel analysis suggested a unidimensional scale structure. The three items had factor loadings of 0.80, 0.82, and 0.86, respectively. A Cronbach's alpha of 0.86 and McDonald's omega of 0.87 were estimated for the three-item scale in this sample. Supplementary Table 2 reports the correlations between the six and the three-item versions and depressiveness as well as anxiety. Compared to the six-item version, the magnitude of the associations between the three-item version and the other self-rated questionnaires were lower, which is to be expected in a shortened version of a questionnaire. The associations, however, remained significant. The newly developed, three-item instrument was further tested using Sample 2.

### Sample characteristics

Participants indicated a mean level of 12.80 (*SD* = 2.71) of perceived social support measured with the F-SozU K-3. More information about the sample can be found in Table 1.

**Table 1:** Sample characteristics.

	Sample 1 (*N* = 2482)				Sample 2 (*N* = 2501)			
	*N / M*	*% / SD*	*alpha*	*omega*	*N / M*	*% / SD*	*alpha*	*omega*
**Sociodemographic information**								
Sex								
* male*	1163	46.9%			1139	45.5%		
* female*	1319	53.1%			1362	54.5%		
Age (*M, SD*)	49.62	18.31			46.01	17.58		
Age groups								
* <25 yr*	254	10.2%			272	10.9%		
* 25–34 yr*	358	14.4%			371	14.8%		
* 34–44 yr*	380	15.3%			420	16.8%		
* 45–54 yr*	441	17.8%			470	18.8%		
* 55–64 yr*	448	18.1%			477	19.1%		
* 65–74 yr*	375	15.1%			314	12.5%		
* >75 yr*	226	9.1%			177	7.1%		
Education								
* High school degree*	537	21.7%			614	24.6%		
* No high school degree*	1935	78.3%			1882	75.4%		
Employment								
* Employment (fulltime or part-time)*	1297	52.3%			1462	59.0%		
* No paid employment*	1185	47.7%			1014	41.0%		
Equivalized income								
* < 1250€*	961	39.9%			654	27.0%		
* 1250 – 2500€*	1342	55.7%			1598	65.8%		
* ≥ 2500€*	106	4.4%			175	7.2%		
Partnership								
* With partner*	1163	46.9%			1135	45.5%		
* Without partner*	1319	53.1%			1359	54.5%		
Number of persons in household (*M, SD*)	2.08	1.10			2.16	1.14		
Living in East Germany	502	20.2%			497	19.9%		
Living in West Germany	1980	79.8%			2004	80.1%		
**Psychological measures**								
Social support (F-SozU K-6; *M, SD*)	24.06	4.56	0.89	0.91	–	–	–	–
Social support (F-SozU K-3; *M, SD*)	12.33	2.51	0.86	0.87	12.80	2.71	0.89	0.89
Depressiveness (PHQ-2; *M, SD*)	2.85	1.21	0.80	0.80	2.89	1.21	0.82	0.82
Anxiety (GAD-2; *M, SD*)	2.88	1.22	0.79	0.79	2.77	1.17	0.81	0.81
Loneliness (GHS-Loneliness; *M, SD*)	–	–	–	–	1.35	0.68	–	–

*Note. M* = Mean; *SD* = Standard deviation.

### Item characteristics

Means (*M*), standard deviations (*SD*) for the items, item difficulties (*P_i_*) and corrected item-total correlation (r_it_) are shown in Table 2. In the full sample, the item difficulties varied between 0.84 and 0.86. Participants scored highest on the item “When I am sick, I can ask friends/relatives to handle important things for me without hesitation”. The corrected item-total correlations achieved very satisfactory values of r*_it_* = 0.78 to r*_it_* = 0.79.

**Table 2:** Means (M), standard deviations (SD), item difficulties (*P_i_*), corrected item-total correlations (*r_it_*), and group differences for the F-SozU K-3 items.

Item			total				With partner				Without partner				Group difference		
	English	German	M	SD	*P_i_*	*r_it_*	M	SD	*P_i_*	*r_it_*	M	SD	*P_i_*	*r_it_*	*t*	*p*	*d*
2	There is someone very close to me whose help I can always count on.	Ich habe einen sehr vertrauten Menschen, mit dessen Hilfe ich immer rechnen kann.	4.29	0.97	0.86	0.79	4.39	0.92	0.88	0.78	4.21	1.01	0.84	0.79	−4.763	**<0.001**	0.19
5	When I am sick, I can ask friends/relatives to handle important things for me without hesitation.	Wenn ich krank bin, kann ich ohne Zögern Freunde/Angehörige bitten, wichtige Dinge für mich zu erledigen.	4.30	0.97	0.86	0.79	4.38	0.95	0.88	0.79	4.23	0.99	0.85	0.79	−3.954	**<0.001**	0.16
6	If I'm very depressed, I know who I can turn to.	Wenn ich mal sehr bedrückt bin, weiß ich, zu wem ich damit ohne weiteres gehen kann.	4.21	1.04	0.84	0.78	4.32	0.95	0.86	0.76	4.12	1.10	0.82	0.80	−4.828	**<0.001**	0.19

In participants with and without a partner, the item difficulties varied between 0.86 to 0.88 and 0.82 to 0.85, respectively. On the item level, there were statistically significant (*p* < 0.001) differences between participants with and without a partner. Participants with a partner scored highest on the item “There is someone very close to me whose help I can always count on”, participants without a partner scored highest on the item “When I am sick, I can ask friends/relatives to handle important things for me without hesitation”. The corrected item-total correlations can be regarded as satisfactory with values between r_it_ = 0.76 and r_it_ = 0.79 in participants with and r_it_ = 0.79 and r_it_ = 0.80 in those without a partner.

### Factorial validity and internal consistency

As the three-item model was just identified, the fit indices could not be calculated (df = 0, CFI = 1.000, TLI = 1.000, RMSEA = 0.000, SRMR = 0.000). However, factor loadings were high (0.85 – 0.86), therefore it can be assumed that all of the three indicators are meaningful indicators for the latent construct. The results can be found in the Table 3. We estimated a Cronbach's alpha of *α* = 0.89 and a McDonald's omega of *ω* = 0.89 for the F-SozU K-3 in this sample, which indicates very good internal consistency.

**Table 3:** Fit indices for testing for measurement invariance with a model for the total sample and models grouped by sex and partnership.

total sample								
	CFI	TLI	RMSEA	SRMR	Factor loadings			
CFA	NA	NA	NA	NA	0.86 0.86 0.85			
**grouped by sex**								
	**CFI**	**TLI**	**RMSEA**	**SRMR**	△ **CFI**	△ **TLI**	△ **RMSEA**	△ **SRMR**
Configural	1.000	1.000	0.000	0.000				
Weak	1.000	1.000	0.006	0.011	0.000	0.000	0.006	0.011
Strong	0.999	0.999	0.026	0.014	−0.001	−0.001	0.020	0.003
Strict	0.993	0.994	0.059	0.016	−0.006	−0.005	0.033	0.002
**grouped by partnership (with or without partner)**								
	**CFI**	**TLI**	**RMSEA**	**SRMR**	△ **CFI**	△ **TLI**	△ **RMSEA**	△ **SRMR**
Configural	1.000	1.000	0.000	0.000				
Weak	0.996	0.989	0.078	0.026	−0.004	−0.011	0.078	0.026
Strong	0.997	0.995	0.055	0.027	0.001	0.006	−0.023	0.001
Strict	0.996	0.997	0.043	0.030	−0.001	0.002	−0.012	0.003

*Note.* We entered NA for the fit measures of the CFA since the three-item model was just identified.

### Factorial invariance

Next, we tested the measurement invariance regarding sex and partnership status (see Table 3). Full invariance for all models can be assumed since there has not been a change in CFI that surpasses the threshold of Δ_CFI_ = 0.01.

### Construct validity

To test the construct validity of the F-SozU K-3, we estimated correlation coefficients with related questionnaires, such as the PHQ-2, the GAD-2, and the GHS loneliness item. The results can be found in Table 4. The directions of the coefficients go in the expected directions: High perceived social support is negatively associated with lower levels of depressiveness (PHQ-2), generalized anxiety (GAD-2), and loneliness (GHS-loneliness).

**Table 4:** Correlation coefficients between the F-SoZu K-3 and other self-rating questionnaires.

Fragebogen	F-SozU K-3	PHQ-2	GAD-2	GHS-Loneliness
F-SozU K-3	1			
PHQ-2	−0.246***	1		
GAD-2	−0.202***	0.686***	1	
GHS-Loneliness	−0.319***	0.388***	0.372***	1

*Note.* Spearman's correlation coefficient was used. ***p < 0.001.

Additionally, we tested for significant differences between various sociodemographic groups, as can be seen in Table 5. We found significantly higher perceived social support in younger versus older participants, females, and those with a partner, as well as currently employed participants. Having a high school degree or not did not yield a significant difference in perceived social support (*p* = 0.143).

**Table 5:** Group differences in perceived social support (F-SozU K-3) of the participants.

	Group 1			Group 2			Significance		
	N	M	SD	N	M	SD	t	p	d
age (14–49 vs. 50–91)	1278	12.91	2.66	1170	12.68	2.77	2.159	**0.031**	−0.09
sex (male vs. female)	1139	12.62	2.75	1362	12.95	2.67	−3.087	**0.002**	0.12
education (high school degree vs. no high school degree)	614	12.94	2.58	1882	12.76	2.75	−1.465	0.143	0.07
partner (yes vs. no)	1135	13.09	2.54	1359	12.56	2.82	−4.990	**0.000**	0.20
employment (yes vs. no)	1014	12.58	2.85	1462	12.95	2.61	3.226	**0.001**	−0.13

*Note. N* = size of the (partial) sample; *M* = mean; *SD* = standard deviation; separate *N, M*, and *SD* for group 1 or group 2; *t* = *t*-value; *p* = *p*-Value; *d* = effect size (Cohen's *d*); varying *N* due to partially missing measured values. “No employment” in our case means that the individual does not pursue any paid work, it therefore includes unpaid civil service work, parental leave, and retirement.

**Fig. 1:**
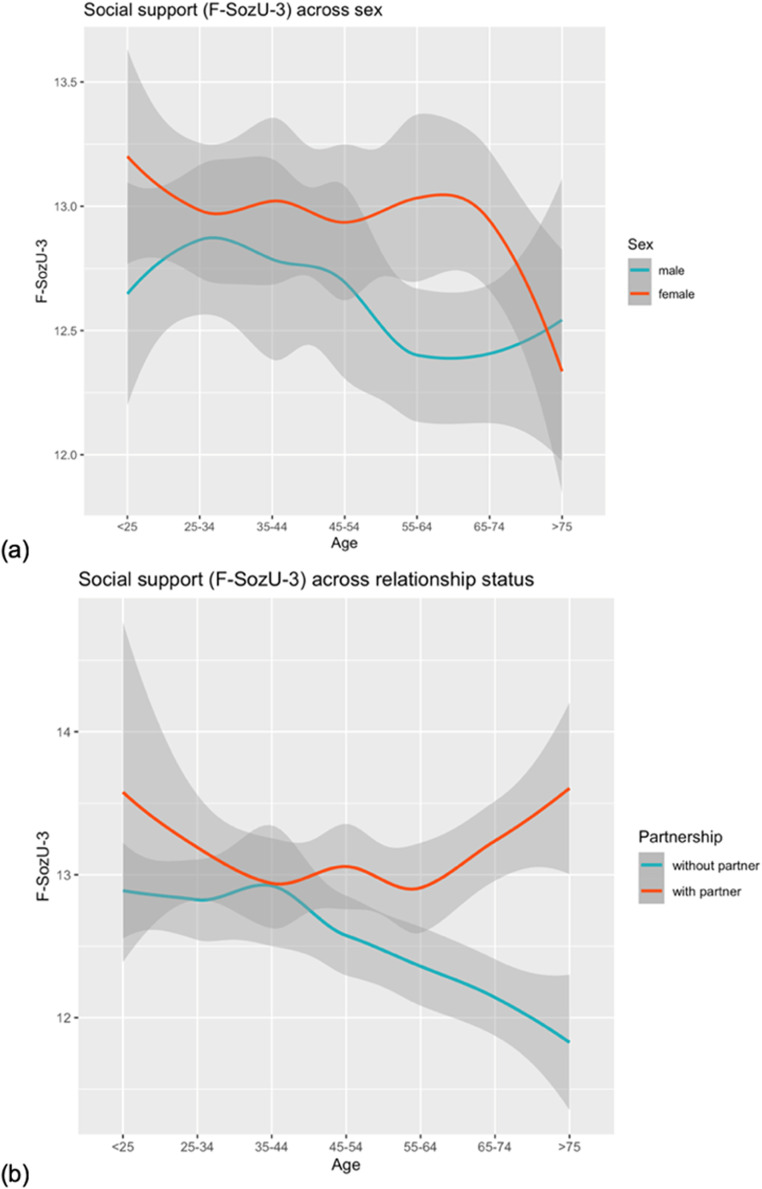
The F-SozU K-3 values depending on age as well as (a) sex (b) partnership status, with a 95% confidence interval.

Finally, we looked at the distribution of the F-SozU K-3 sum score according to sex and age (see Fig. 1a), as well as according to partnership status and age (see Fig. 1b). Additional results from a two-way ANOVA with the factors sex and age groups showed a significant main effect of sex (*F* (2487) = 6.695, *p* = 0.008), a nonsignificant effect for age groups (*F* (2487) = 1.425, *p* = 0.201), and for the interaction of sex and age groups (*F* (2487) = 1.268, *p* = 0.269). The two-way ANOVA with the factors sex and partnership status, however yielded significant results for all measured effects (sex: *F* (2490) = 9.447, *p* = 0.002; partnership status: *F* (2490) = 27.162, *p* < 0.001; interaction: *F* (2490) = 4.392, *p* = 0.036). Therefore, we opted to provide norm values according to partnership status and sex in addition to the general norms.

### Norm values

Lastly, we calculated norm values for the F-SozU K-3, as can be seen in Table 6. Since the sum score of the F-SozU K-3 deviated from the normal distribution, the sum score was reported using percentiles.

**Table 6:** Percentiles for the F-SozU K-3 according to sex and partnership.

		Men		Women	
F-SozU sum score	General	With partner	Without partner	With partner	Without partner
	*N* = 2501	*N* = 536	*N* = 600	*N* = 599	*N=* 759
3	1	1	1	1	1
4	1	1	1	1	1
5	2	1	3	2	2
6	3	2	5	2	4
7	5	4	7	4	6
8	8	7	11	6	9
9	15	13	20	12	15
10	20	18	25	16	19
11	25	23	32	21	25
12	38	33	48	30	38
13	46	41	56	40	46
14	56	54	65	51	54
15	100	100	100	100	100

*Note.* Normative data are presented as F-SozU-3 sum scores with corresponding percentiles. Percentiles are shown for the total sample and for subsamples based on sex and partnership.

## Discussion

The Social Support Questionnaire (F-SozU) and its brief versions (F-SozU K-22, F-SozU K-14, F-SozU K-6) are commonly used and have been proven to be valid instruments to measure perceived social support. The purpose of the present study was the development and validation of a brief version compromising three items, which can be used as a screening instrument for the measurement of perceived social support in large scale surveys, by shortening the F-SozU K-6. The F-SozU K-3 showed a good internal consistency with α = 0.86 and 0.89, as well as ω = 0.87 and 0.89 across both samples. Although the model was just identified due to its three-item nature and thus could not be tested using a confirmatory factor analysis, we found that the item loadings were consistently high and loaded onto one factor, indicating that these items were good indicators for the brief three-item form of the F-SozU.

Therefore, the F-SozU K-3 presents a reliable, valid and economical instrument to assess perceived social support in the general German-speaking population. Further, strict measurement invariance across sex and age was given, which indicates the ability to compare those groups.

Replicating well-established associations of perceived social support with depression symptoms, symptoms of generalized anxiety and loneliness of the longer versions (Fydrich et al., 2007, 2009), we attested construct validity of the F-SozU K-3. It has to mentioned, however, that compared to the F-SozU K-6 these associations were smaller in size, which is to be expected given the reduction of items.

Additionally, we found that younger participants (< 50 years), women, participants with a partner and paid employment scored significantly higher, which is consistent with previous findings (Fydrich et al., 2009). Interestingly, the means of the F-SOZU K-3 items were higher in the second sample than in the first sample. This might be due to younger age, higher educational, employment, and income levels of the second sample, which is consistent with the positive associations with these factors. Finally, norm values were reported across sex and partnership.

### Limitations

Using data of two representative German population surveys is a great strength of the present study. However, our results should be interpreted with caution. Though our findings are representative for the German general population, they might not be comparable to cultures outside of the Western world. Therefore, we suggest to replicate our findings with data from countries with different cultural backgrounds. Additionally, the psychometric properties reported in this study might differ from those found in other samples, such as, for example, in clinical populations. It also has to be mentioned that both samples used in this sample were surveyed before the COVID-19 pandemic. Severe restrictions on physical social contact might have impacted the social support and therefore increased the risk of social isolation.

## Conclusion

Summed up, the newly developed F-SozU K-3 can be used as a reliable and valid screening tool to measure perceived social support in large scale surveys, where an economical assessment is of key importance.

## Availability of data and materials

The datasets supporting the conclusions of this article are available from the corresponding author on reasonable request.

## Funding

This research did not receive any specific grant from funding agencies in the public, commercial, or not-for-profit sectors.

## Research support

This research received no external financial or non-financial support.

## Relationships

There are no additional relationships to disclose.

## Patents and intellectual property

There are no patents to disclose.

## Other activities

There are no other activities to disclose.

## CRediT authorship contribution statement

**Julia Petersen:** Methodology, Formal analysis, Visualization, Writing – original draft. **Anna C. Reinwarth:** Writing – original draft. **Manfred E. Beutel:** Writing – review & editing, Supervision. **Elmar Brähler:** Resources, Writing – review & editing, Supervision. **Oliver Decker:** Writing – review & editing.

## Declaration of competing interest

The authors declare that they have no competing interests.

## Appendix Supplementary materials

Image, application 1
