# Supplementary material for: Development and validation of a brief three-item form of the perceived social support questionnaire (F-SozU K-3)
Source: Int J Clin Health Psychol. 2024 Aug 30;24(3):100496. doi: 10.1016/j.ijchp.2024.100496 (PMC11402393; doi:10.1016/j.ijchp.2024.100496)
Supplement: Supplementary file 1 [file mmc1.docx]

**Supplementary Table 1.** Means (M), standard deviations (SD), item difficulties (*P_i_*), corrected item-total correlations (*r_it_*), and group differences for the F-SozU K-6 items.

| **Item** | | | **total** | | | | **With partner** | | | | **Without partner** | | | | **Group difference** | | |
| --- | --- | --- | --- | --- | --- | --- | --- | --- | --- | --- | --- | --- | --- | --- | --- | --- | --- |
|  | **English** | **German** | **M** | **SD** | ***P_i_*** | ***r_it_*** | **M** | **SD** | ***P_i_*** | ***r_it_*** | **M** | **SD** | ***P_i_*** | ***r_it_*** | ***t*** | ***p*** | ***d*** |
| 1 | I receive a lot of understanding and security from others. | Ich erfahre von anderen viel Verständnis und Geborgenheit. | 3.73 | 0.93 | 0.75 | 0.67 | 3.94 | 0.86 | 0.79 | 0.64 | 3.55 | 0.95 | 0.71 | 0.67 | -10.618 | **0.000** | 0.42 |
| 2 | There is someone very close to me whose help I can always count on. | Ich habe einen sehr vertrauten Menschen, mit dessen Hilfe ich immer rechnen kann. | 4.17 | 0.94 | 0.83 | 0.74 | 4.40 | 0.80 | 0.88 | 0.70 | 3.96 | 1.01 | 0.79 | 0.75 | -11.898 | **0.000** | 0.47 |
| 3 | If I need to, I can borrow something from friends or neighbors without any problems. | Bei Bedarf kann ich mir ohne Probleme bei Freunden oder Nachbarn etwas ausleihen. | 3.98 | 0.94 | 0.79 | 0.66 | 4.09 | 0.90 | 0.82 | 0.62 | 3.88 | 0.96 | 0.76 | 0.69 | -5.617 | **0.000** | 0.23 |
| 4 | I know several people with whom I like to do things. | Ich kenne mehrere Menschen, mit denen ich gerne etwas unternehme. | 4.02 | 0.95 | 0.80 | 0.66 | 4.10 | 0.88 | 0.82 | 0.68 | 3.96 | 0.99 | 0.79 | 0.66 | -3.688 | **0.000** | 0.15 |
| 5 | When I am sick, I can ask friends/relatives to handle important things for me without hesitation. | Wenn ich krank bin, kann ich ohne Zögern Freunde/Angehörige bitten, wichtige Dinge für mich zu erledigen. | 4.10 | 0.92 | 0.82 | 0.78 | 4.27 | 0.82 | 0.85 | 0.74 | 3.95 | 0.98 | 0.79 | 0.79 | -8.917 | **0.000** | 0.35 |
| 6 | If I’m very depressed, I know who I can turn to. | Wenn ich mal sehr bedrückt bin, weiß ich, zu wem ich damit ohne weiteres gehen kann. | 4.06 | 0.97 | 0.81 | 0.77 | 4.22 | 0.90 | 0.84 | 0.73 | 3.92 | 1.00 | 0.78 | 0.78 | -7.962 | **0.000** | 0.32 |

**Supplementary Table 2. Correlation coefficients between the F-SozU K-6, the F-SoZu K-3 and other self-rating questionnaires**

| **Fragebogen** | **F-SozU K6** | **F-SozU K-3** | **PHQ-2** | **GAD-2** |
| --- | --- | --- | --- | --- |
| F-SozU K-6 | 1 |  |  |  |
| F-SozU K-3 | 0.940*** | 1 |  |  |
| PHQ-2 | -0.246*** | -0.210*** | 1 |  |
| GAD-2 | -0.220*** | -0.192*** | 0.679*** | 1 |

*Note.* Spearman’s correlation coefficient was used. ****p* < 0.001
